# Mitochondrial Quality Control and Metabolic Memory Phenomenon Associated with Continued Progression of Diabetic Retinopathy

**DOI:** 10.3390/ijms24098076

**Published:** 2023-04-29

**Authors:** Renu A. Kowluru, Kumari Alka

**Affiliations:** Department of Ophthalmology, Visual and Anatomical Sciences, Wayne State University, Detroit, MI 48201, USA

**Keywords:** diabetic retinopathy, metabolic memory, mitochondria, mitochondrial dynamics, mitophagy

## Abstract

Diabetic retinopathy continues to progress even when hyperglycemia is terminated, suggesting a ‘metabolic memory’ phenomenon. Mitochondrial dysfunction is closely associated with the development of diabetic retinopathy, and mitochondria remain dysfunctional. Quality control of mitochondria requires a fine balance between mitochondrial fission–fusion, removal of the damaged mitochondria (mitophagy) and formation of new mitochondria (biogenesis). In diabetes, while mitochondrial fusion protein (Mfn2) is decreased, fission protein (Drp1) is increased, resulting in fragmented mitochondria. Re-institution of normal glycemia fails to reverse mitochondrial fragmentation, and dysfunctional mitochondria continue to accumulate. Our aim was to investigate the direct effect of regulation of the mitochondrial fusion process during normal glycemia that follows a high glucose insult on mitochondrial quality control in the ‘metabolic memory’ phenomenon. Human retinal endothelial cells, incubated in 20 mM glucose for four days, followed by 5 mM glucose for four additional days, with or without the Mfn2 activator leflunomide, were analyzed for mitochondrial fission (live cell imaging), mitophagy (flow cytometry and immunofluorescence microscopy), and mitochondrial mass (mitochondrial copy numbers and MitoTracker labeling). Mitochondrial health was determined by quantifying mitochondrial reactive oxygen species (ROS), respiration rate (Seahorse XF96) and mitochondrial DNA (mtDNA) damage. Addition of leflunomide during normal glucose exposure that followed high glucose prevented mitochondrial fission, facilitated mitophagy and increased mitochondrial mass. Glucose-induced decrease in mitochondrial respiration and increase in ROS and mtDNA damage were also prevented. Thus, direct regulation of mitochondrial dynamics can help maintain mitochondrial quality control and interfere with the metabolic memory phenomenon, preventing further progression of diabetic retinopathy.

## 1. Introduction

Retinopathy is one of the most feared complications of diabetes, and remains the leading cause of acquired blindness in working-age adults. Although many systemic factors are thought to contribute to its development, hyperglycemia remains the main instigator. The landmark Diabetes Control and Complications Trial (DCCT) and Epidemiology of Diabetes Interventions and Complications (EDIC) studies documented that the progression of retinopathy does not halt, even when intensive glucose control was maintained in patients that were on a conventional regimen during the DCCT [1,2,3]. These studies demonstrated the importance of early intensive control and have shown that the benefits of early control continue beyond the period of its institution; 30 years after the start of the DCCT, patients in prior intensive control had better visual function compared to those in conventional control, implying a ‘metabolic imprinting/memory’ phenomenon [4,5,6]. This metabolic memory phenomenon is also demonstrated in the experimental models of diabetic retinopathy; chemically induced diabetic dogs and rodents have shown retinal histopathology initiated during poor glycemic control to continue even when poor glycemic control is reversed by good glycemic control [7,8], and retinal vascular cells incubated in normal glucose continue to undergo apoptosis after a period of high glucose [9], a phenomenon that precedes the development of histopathology [10]. However, the exact molecular mechanism of the metabolic memory phenomenon remains to be elucidated.

Mitochondria are the key regulators of cell fate, controlling cell survival via producing ATP and cell death via releasing pro-apoptotic cytochrome C in the cytosol [11]. Dysfunctional/damaged mitochondria produce free radicals that impair their functional, structural and genomic stability; retinal mitochondria become dysfunctional, their DNA (mtDNA) is damaged, and biogenesis and removal of the damaged mitochondria are impaired in diabetic retinopathy [12,13]. Mitochondrial quality control is maintained by a balance between mitochondrial fission–fusion, mitophagy and biogenesis; mitochondria undergo constant fusion–fission to maintain their number, size and shape, and the damaged mitochondria are removed by mitophagy. Mitochondrial dynamics are mediated by a group of small GTPases; while dynamin-related protein-1 (Drp1) is critical for the fission process, mitofusin 1 and 2 (Mfn1 and Mfn2) regulate outer membrane fusion and optic atrophy-1 helps in the fusion of the mitochondrial inner membrane [14]. Mitochondrial biogenesis generates new mitochondria in response to mitochondrial damage and increased energy demand; any imbalance in mitochondrial dynamics, mitophagy or biogenesis results in dysfunctional mitochondria and increases ROS accumulation [14,15]. In diabetic retinopathy, due to the inhibition of Mfn2 and activation of Drp1, mitochondrial fragmentation is increased, and removal of the damaged mitochondria and mitochondrial biogenesis becomes suboptimal. Re-institution of normal glycemia after a period of hyperglycemia does not provide any benefit to the damaged mitochondria, and mitochondrial dysfunction continues [16,17]. Mitochondrial fusion–fission remains imbalanced, sustained accumulation of damaged/fragmented mitochondria continues to produce free radicals, and retinal capillary cell apoptosis continues to progress [17,18]}. Direct intervention of good glycemia with DNA methylation inhibitors or fission protein inhibitors following hyperglycemia prevents the accumulation of fragmented mitochondria [17,19]. However, the direct effect of the regulation of dynamics on mitochondrial quality control remains unclear.

The aim of this study was to investigate the direct effect of the regulation of the mitochondrial fusion process on mitochondrial quality control during normal glycemia following high glucose insult in terms of the metabolic memory phenomenon. Using human retinal endothelial cells (HRECs), we investigated the direct effect of leflunomide [20], an Mfn2 activator, on continued impairment in mitochondrial dynamics, removal of damaged mitochondria and biogenesis of new mitochondria during normal glucose exposure that followed a high glucose insult.

## 2. Results

**Figure 1:**
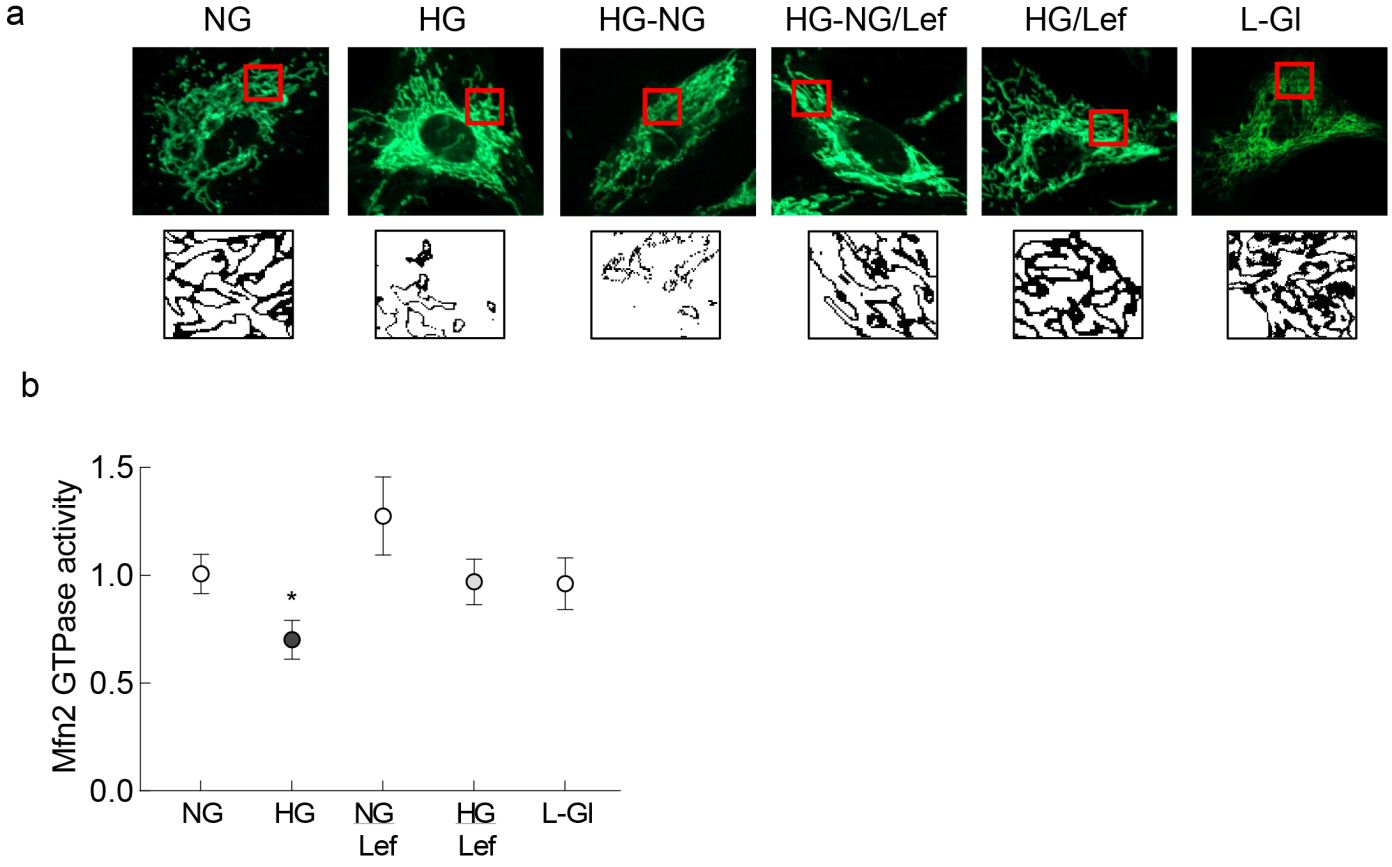
Supplementation with leflunomide during normal glucose (5 mM D-glucose) exposure that followed high glucose (20 mM D-glucose) and mitochondrial fragmentation. (**a**) Live cell microscopy was performed to evaluate mitochondrial morphology using MitoTracker Green. The panel below the MitoTracker Green panel shows the bare outlines of the area inside the red box. Each image is representative of 4–6 cells/group, repeated in three or more cell preparations. (**b**) Graph representing the effect of leflunomide on GTPase activity of Mfn2. NG and HG = 5 mM or continuous 20 mM D-glucose, respectively; HG-NG and HG-NG/Lef = 20 mM D-glucose for four days followed by 5 mM glucose, without or with leflunomide, respectively; NG/Lef and HG/Lef = 5 mM or 20 mM D-glucose, with leflunomide; L-Gl = 20 mM L-glucose. * *p* < 0.05 vs. NG.

Previous work has shown that reversal of hyperglycemia with normal glycemia has no beneficial effect on mitochondrial dynamics in retinal vasculature, and mitochondria continue to be dysfunctional and fragmented [17,19]. Here, we show that the supplementation of normal glucose (5 mM D-glucose, NG) incubation with an activator of the fusion enzyme Mfn2, leflunomide (HG-NG/Lef group), prevented mitochondrial fission following four days of high glucose (20 mM D-glucose, HG). The elongated mitochondrial structure in these groups was similar to that observed in cells in normal glucose or in high glucose supplemented with leflunomide (HG/Lef), and was different from the fragmented mitochondria in continuous high glucose or four days of high glucose followed by four days of normal glucose (HG-NG group). HRECs in 20 mM L-glucose (L-Gl group) had similar mitochondrial morphology as in the NG group (Figure 1a). Figure 1b is included to show that leflunomide prevents glucose-induced decrease in the GTPase activity of Mfn2.

**Figure 2:**
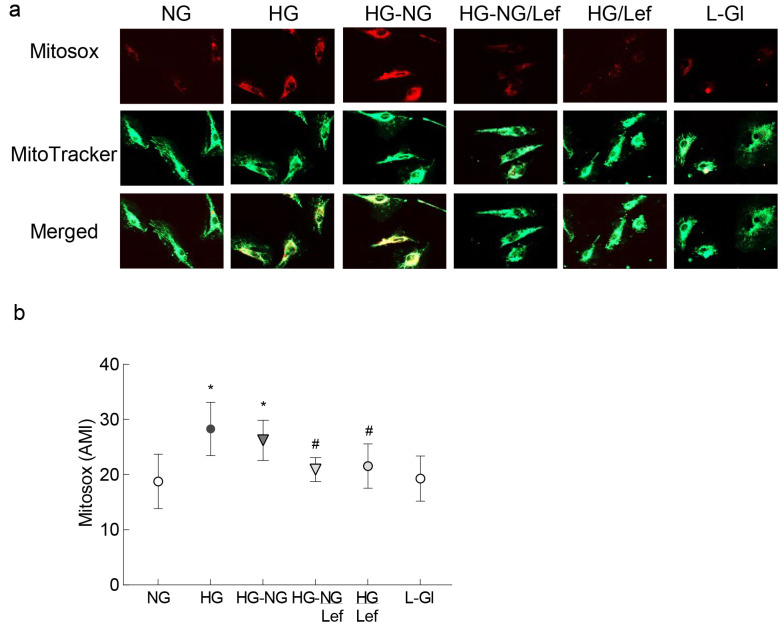
Mitochondrial ROS and regulation of mitochondrial fusion. (**a**) Live cells were stained with MitoSOX Red and MitoTracker Green and imaged under a Zeiss Apotome using 40× objective. (**b**) Graph showing arithmetic mean intensity (AMI), calculated from 3 coverslips/group using the Zeiss colocalization software module, with 6–8 cell/coverslip, and repeated in 2–3 cell preparations. NG and HG = 5 mM or 20 mM continuous D-glucose, respectively; HG-NG and HG-NG/Lef = 20 mM D-glucose for four days followed by 5 mM glucose, without or with leflunomide, respectively; HG/Lef = 20 mM D-glucose+ leflunomide; L-Gl = 20 mM L-glucose. * *p* < 0.05 vs. NG and # *p* < 0.05 vs. HG.

Mitochondrial fragmentation results in their dysfunction [14,15]; the effect of regulation of dynamics on mitochondrial ROS was quantified. As expected, four days of normal glucose incubation following four days of high glucose exposure did not prevent an increase in mitochondrial ROS levels, as evidenced by increase in MitoSOX staining, but the addition of leflunomide during the reversal phase ameliorated sustained increase in mitochondrial ROS (Figure 2a) and the values in the HG-NG groups were significantly higher compared to those in the HG-NG/Lef group (Figure 2b).

**Figure 3:**
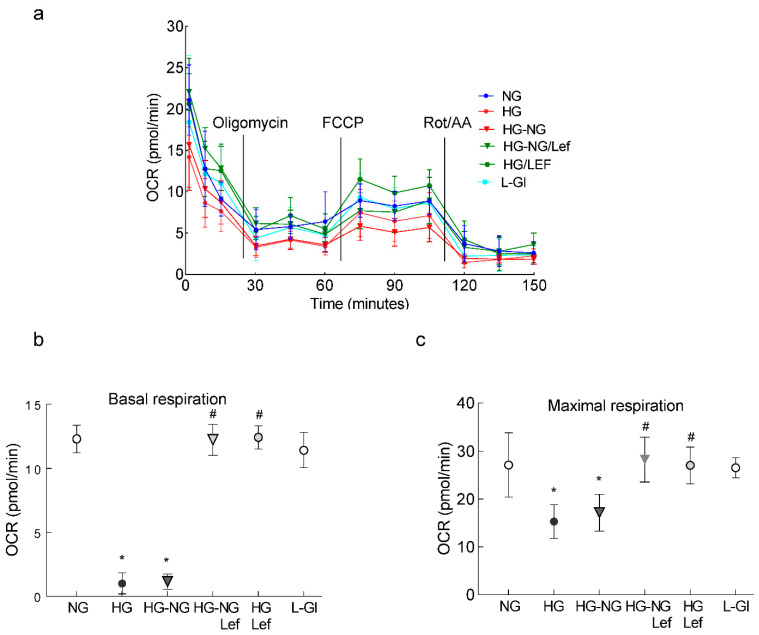
Mitochondrial respiration and leflunomide supplementation during normal glucose following high glucose. (**a**) Oxygen consumption rate (OCR), (**b**) basal respiration and (**c**) maximal respiration rates were measured in HRECs using a Seahorse XF analyzer and a Seahorse XF Cell Mito Stress Test Kit. Measurements were repeated 3–5 times, with each measurement containing 5 or more wells/group; the results in the graphs are presented as mean ± SD. NG = 5 mM D-glucose; HG and HG/Lef = 20 mM D-glucose without and with leflunomide, respectively; HG-NG and HG-NG/Lef = 20 mM D-glucose for four days followed by 5 mM D-glucose without or with leflunomide, respectively; L-Gl = 20 mM L-glucose; FCCP = carbonyl cyanide p-trifluoro methoxyphenylhydrazone; Rot/AA = rotenone/antimycin A. * *p* < 0.05 vs. NG and # *p* < 0.05 vs. HG.

In accordance with mitochondrial ROS, leflunomide supplementation during the four days of normal glucose incubation that followed four days of high glucose also ameliorated mitochondrial dysfunction, as determined using a Seahorse XF96 instrument tracking the oxygen consumption rate (OCR) following injections of the selected inhibitors/activators (Figure 3). Compared to cells in normal glucose, high glucose significantly decreased the basal respiration rate, measured before injecting any compounds to regulate complex activities, and this decrease was not reversed after termination of high glucose (HG-NG group; Figure 3b). Oligomycin injection further decreased OCR in the HG and HG-NG groups. Addition of an uncoupler carbonylcyanide-p-trifluoromethoxyphenylhydrazone (FCCP) to simulate physiological energy demand increased energy demand in the NG group, but in the HG and HG-NG groups this effect was markedly blunted, and the maximal respiration rate, measured after FCCP injection, was also significantly reduced in these two groups (Figure 3c). However, supplementation with leflunomide during the continuous high glucose incubation (HG/Lef group) or during normal glucose following high glucose (HG-NG/Lef groups) ameliorated the decrease in mitochondrial respiration, and basal and maximal respiration rates in the HG-NG/Lef and HG/Lef groups were similar to those obtained from cells in the NG and L-Gl groups.

**Figure 4:**
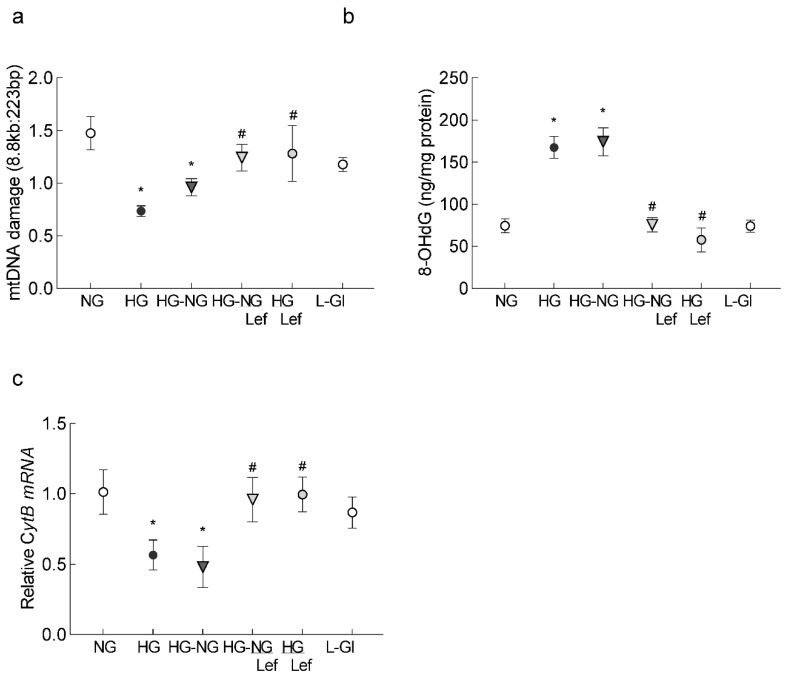
Leflunomide supplementation and mtDNA damage. (**a**) Oxidatively modified DNA, 8-OHdG, was measured in the mitochondria. (**b**) Extended-length PCR was performed on the genomic DNA; the graph represents the ratio of the long amplicon to the short amplicon. (**c**) Gene transcripts of *CytB*, quantified by qRT-PCR using *β-actin* as the housekeeping gene. The values are represented as mean ± SD from 3–4 different cell preparations, using 2–3 samples/group, and each sample quantified in duplicate. NG and HG = 5 mM and 20 mM D-glucose, respectively; HG-NG and HG-NG/Lef = 20 mM D-glucose followed by 5 mM glucose without or with leflunomide, respectively; HG/Lef = 20 mM D-glucose with leflunomide; L-Gl = 20 mM L-glucose. * *p* < 0.05 compared to NG and # *p* < 0.05 compared to HG.

Since sustained increase in ROS oxidatively modifies DNA and our previous studies have shown that mtDNA continues to be damaged even when hyperglycemia is terminated [21], the effect of the regulation of mitochondrial dynamics on DNA damage was investigated. Regulation of mitochondrial dynamics by leflunomide during the reversal phase prevented an increase in 8-hydroxy-2-deoxyguanosine (8-OHdG) levels and damage to the mtDNA (Figure 4a,b). Consistent with mtDNA damage, gene transcripts of mtDNA-encoded *CytB* were significantly higher in the HG-NG/Lef group compared to the HG-NG or HG groups, suggesting restoration of mtDNA transcription (Figure 4c). Cells in 20 mM L-glucose had comparable values to those obtained from cells in 5 mM D-glucose.

**Figure 5:**
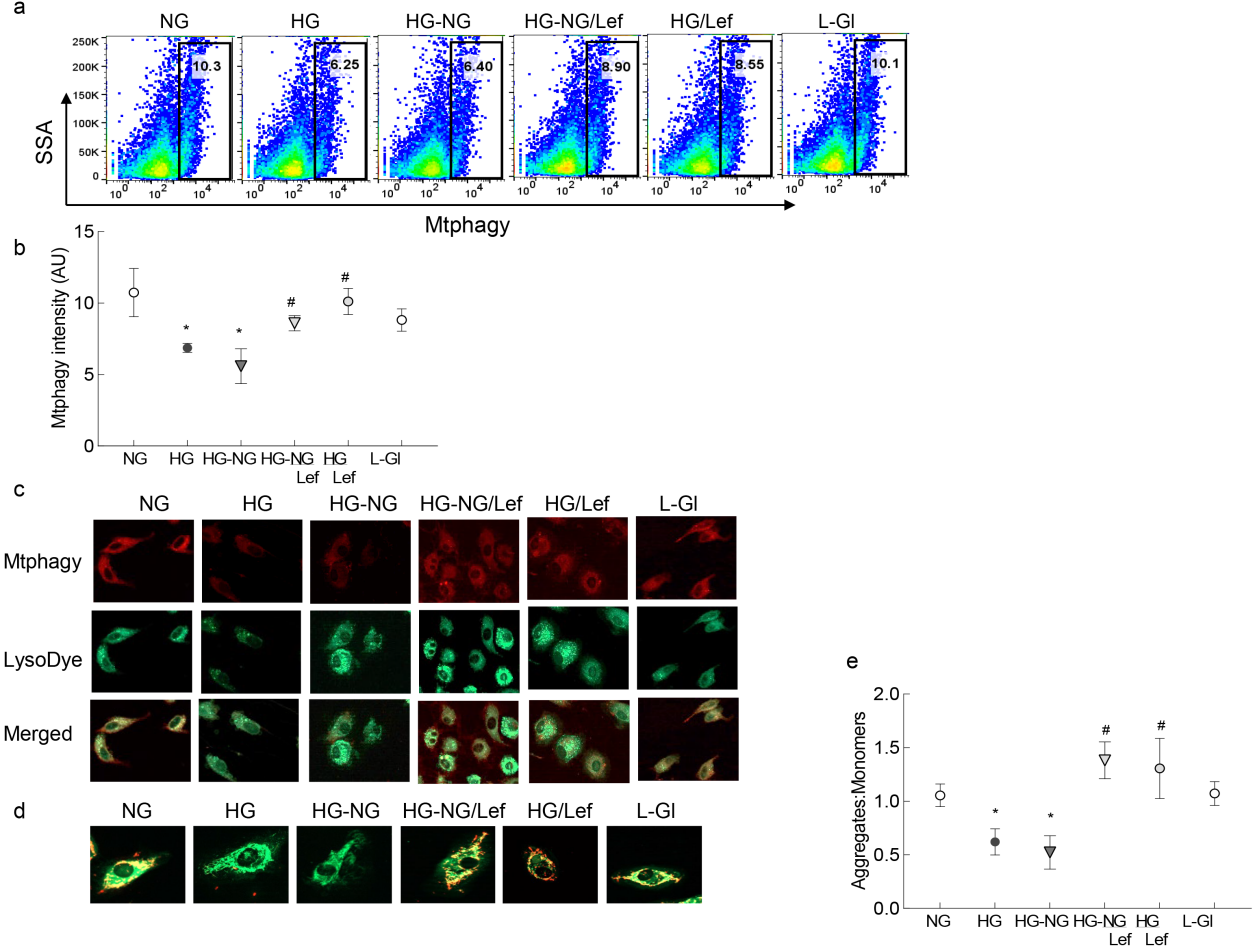
Effect of the regulation of mitochondrial fusion on mitophagy. (**a**) Mitophagy flux was analyzed by staining HRECs with Mtphagy Dye and measuring the intensity of Mtphagy Dye with flow cytometry. (**b**) Fluorescence intensity of the Mtphagy Dye. (**c**) Representative image of HRECs stained with Mtphagy Dye (red) and Lyso Dye (green) and visualized under a Zeiss Apotome fluorescence microscope at 20× magnification. (**d**) Representative image of cells stained with JC-1, showing green fluorescent monomers and red fluorescent aggregates. (**e**) Ratio of aggregates to monomers. Each measurement was made with three or more different cell preparations. NG and HG = 5 mM and 20 mM D-glucose, respectively; HG-NG and HG-NG/Lef = 20 mM D-glucose followed by 5 mM glucose without or with leflunomide, respectively; HG/Lef = 20 mM D-glucose with leflunomide; L-Gl = 20 mM L-glucose. * *p* < 0.05 compared to NG and # *p* < 0.05 compared to HG.

Mitochondrial dynamics are intimately associated with the removal of damaged mitochondria [22]; as expected, reversal of high glucose insult had no effect on mitophagy and it continued to be impaired in the HG-NG group, but leflunomide supplementation during normal glucose exposure facilitated the mitophagy process. The values in the HG-NG/Lef and HG/Lef groups were significantly higher compared to those in the HG-NG or HG group and were similar to those obtained in the NG or L-Gl groups (Figure 5a,b). Consistent with mitophagy flux, glucose-induced decreased staining of cells with Mtphagy Dye and LysoDye was ameliorated by leflunomide supplementation during the reversal phase (Figure 5c). Furthermore, in the HG-NG and HG groups, mitochondrial potential was also impaired with decreased J-aggregates, and leflunomide supplementation prevented sustained impairment in the membrane potential. Cells in 5mM D-glucose or 20 mM L-glucose had similar values, and these values were significantly different from cells in the HG or HG-NG groups (Figure 5c).

**Figure 6:**
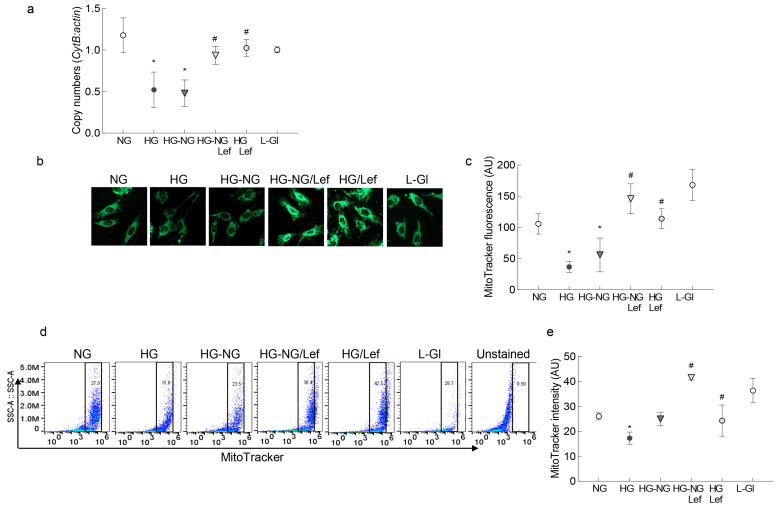
Mfn2 regulation and mitochondrial biogenesis. (**a**) Ratio of the gene transcripts of mtDNA-encoded *CytB* and nuclear DNA-encoded *β-actin*, quantified in the genomic DNA. (**b**) Representative image of cells stained with MitoTracker Green and visualized using a 20× objective. (**c**) Fluorescence intensity of MitoTracker Green (arbitrary units). (**d**) Fluorescence intensity of MitoTracker Green was measured in a flow cytometer, and (**e**) represents the fluorescence intensity of MitoTracker Green (arbitrary units). Values in the graphs are represented as mean ± SD from 3–5 different cell preparations. NG = 5 mM D-glucose; HG and HG/Lef = 20 mM D-glucose, without or with Leflunomide, respectively; HG-NG and HG-NG/Lef = 20 mM D-glucose followed by 5 mM glucose without or with leflunomide, respectively; L-Gl = 20 mM L-glucose. * *p* < 0.05 vs. NG and # *p* < 0.05 vs. HG.

Mitochondrial biogenesis is another major component of the mitochondrial quality control process [23]; in accordance with the continued progression of impaired mitochondrial dynamics and mitophagy, the reversal of high glucose with normal glucose provided no benefit to mitochondrial biogenesis. Mitochondrial copy numbers, as measured by the ratio of *CytB* and *β-actin* gene transcripts in the genomic DNA and by mitochondrial mass, was measured by MitoTracker intensity using immunofluorescence microscopy or flow cytometry. It remained subnormal in the HG-NG group. The values in the HG-NG and HG groups were not different from each other, but were significantly lower than those obtained from the NG or L-Gl groups (Figure 6a–c). However, leflunomide addition ameliorated the sustained decrease in mitochondrial copy numbers, and the values obtained from the HG-NG/Lef and HG/Lef groups were significantly different from those obtained from the HG-NG or HG groups but were comparable to the values in the NG and HG/Lef groups (Figure 6a–c).

## 3. Discussion

Diabetic retinopathy, a slow-progressing disease, is one of the most devastating complications of diabetes. Hyperglycemia is considered the main instigator in its development, but it can progress even after diabetic patients achieve good glycemic control, suggesting a legacy effect. The ongoing effect of prior hyperglycemia depends upon the severity of the hyperglycemia and the duration of this exposure. Thirty years after the start of the DCCT, metabolic memory continues to influence outcomes; EDIC studies have shown visual function differences in patients under prior intensive control and under conventional control [4]. Our previous work using experimental models has shown that mitochondrial dysfunction plays a major role in the development of diabetic retinopathy and in the metabolic processes associated with its continued progression. Mitochondria are very dynamic and constantly undergo regulated fusion and fission to determine their size, shape, numbers and function. Fusion of the damaged mitochondria with healthy mitochondria dilutes molecular damage, but the damaged/dysfunctional giant mitochondria that are seen in diseased cells do not fuse properly and are eliminated by mitophagy; biogenesis makes new mitochondria [15,24]. However, an imbalance in fusion–fission also compromises mitophagy and removal of the damaged mitochondria is impaired, resulting in continuous production of free radicals [25,26]. While mitochondrial fission is increased, their fusion is decreased in diabetes, and reversal of hyperglycemia does not provide any benefit to the damaged mitochondria [16,17]. Here, we showed that activation of Mfn2 by a small-molecule activator of the fusion process during normal glucose exposure that follows high glucose insult, in addition to preventing mitochondrial fission, also facilitates removal of damaged mitochondria and increases biogenesis of new mitochondria, suggesting a beneficial effect of the regulation of mitochondrial dynamics on overall mitochondrial quality control.

Mitochondria can rapidly adjust to meet the metabolic needs of the cell via mitochondrial biogenesis and fusing together to meet an increased energy demand, or undergoing fission and mitophagy when the energy demand is decreased. Mitochondrial dynamics maintain bioenergetic health by fusing to maintain a healthy mitochondrial population and by dividing to separate the damaged mitochondria from healthy networks; the dysfunctional mitochondria are removed by mitophagy [27]. In mild stress conditions, mitochondria hyperfuse to generate more ATP temporarily, blocking mitophagy and apoptosis [15]. Furthermore, in normal physiological conditions, mild stress-induced mitochondrial fission is also associated with pro-survival mitophagy, which clears the damaged mitochondria and prevents continuous production of free radicals [22]. However, when stress levels are high, e.g., disease states, damaged/fragmented mitochondria result in accelerated apoptosis [28]. Here, our results show that the regulation of mitochondrial fusion by leflunomide ameliorates mitochondrial dysfunction and increases mitophagy, helping in the removal of damaged mitochondria in addition to preventing mitochondrial fission. In support, reversal of hyperglycemia provides no benefit to cell apoptosis, and accelerated capillary cell apoptosis continues to produce degenerative capillaries in the retinal vasculature [18].

Dysfunctional mitochondria continue to harbor damaged mtDNA and produce free radicals, and reversal of high glucose insult with normal glucose fails to provide any relief to the accumulation of free radicals and mtDNA damage [21]. Our data demonstrate that the direct activation of the mitochondrial fusion process during a normal glucose phase that follows high glucose stress prevents an increase in free radicals and accumulation of oxidized/damaged mtDNA in addition to regulating mitochondrial dynamics. The transcription of mtDNA-encoded genes, important for the functioning of the electron transport chain, is improved. Furthermore, regulation of mitochondrial dynamics also results in preventing mitochondrial stress, and basal and maximal respiration levels become similar to those obtained from cells in continuous normal glucose, suggesting an overall improvement in mitochondrial quality/stability.

Mitochondrial dynamics and biogenesis are reciprocally coupled. In response to energy demand and stress conditions, the cell produces new mitochondria from the ones already existing and increases mitochondrial mass; changes in mitochondrial mass, size, number, morphology and distribution occur in a dynamic manner [23,24]. In normal physiological conditions, mitochondrial fission, by increasing the opening of the mitochondrial permeability transition pore channels, increases mitochondrial biogenesis [29]. However, in diabetic retinopathy, mitochondrial biogenesis is also impaired, and mitochondrial mass is decreased significantly. This hyperglycemia-induced decrease in mitochondrial biogenesis does not pause, even when hyperglycemia is terminated. The presented results clearly demonstrate that regulation of mitochondrial dynamics also improves the decrease in mitochondrial mass and in mitochondrial numbers, when supplemented during the normal glucose exposure of HRECs that had a prior high-glucose exposure.

We used leflunomide as an activator of mitochondrial fusion; however, we recognize that its use in rheumatoid arthritis is considered through inhibition of pyrimidine biosynthesis, and it can affect other biological pathways, including signaling through β-catenin [30,31]. The possibility of mechanisms other than mitochondrial fusion, including amelioration of cytokine production and oxidative stress [32], contributing to mitochondrial quality control by leflunomide supplementation cannot be ruled out. However, leflunomide is shown to activate mitofusins and promote mitochondrial fusion [33,34], and oral leflunomide enhances Mfn2 expression and cell survival [35], suggesting that the beneficial effects of leflunomide on mitochondrial quality control observed in the present study are possibly mediated by the regulation of mitochondrial fusion.

As mentioned above, mitochondrial dynamics involve two opposing processes, mitochondrial fusion and fission, and a fine balance between these two opposing processes is important in maintaining mitochondrial homeostasis. However, mitochondrial quality control is critical for cell survival, and a constant balance is essential between mitochondrial fission–fusion, mitophagy and biogenesis [36]. We have shown that impaired mitochondrial dynamics and biogenesis do not benefit from the termination of hyperglycemic insult, and the damaged mitochondria continue to accumulate [16,17]. The present study clearly demonstrates that direct regulation of mitochondrial fusion can help maintain mitochondrial quality control and prevent accumulation of damaged mitochondria by increasing their removal and facilitating biogenesis of the new mitochondria. Thus, strategies targeted at maintaining mitochondrial dynamics have the potential to prevent the development of diabetic retinopathy by maintaining mitochondrial homeostasis, and if instituted during good glycemic control could also interfere with the metabolic memory phenomenon, preventing further progression of diabetic retinopathy.

## 4. Methods and Materials

Human retinal endothelial cells, HRECs (cat. no. ACBRI 181, Cell Systems Corp., Kirkland, WA, USA) were cultured in an environment of 95% O_2_ and 5% CO_2_ in Dulbecco’s modified Eagle medium (DMEM, cat. no. D5523; Sigma-Aldrich Corp., St. Louis, MO, USA) supplemented with 20 μg/mL endothelial cell growth supplement, 12% heat-inactivated fetal bovine serum and 1% each insulin-, transferrin- and selenium-Glutamax and antibiotic/antimycotic. HRECs from the 6th to 8th passage were incubated in 5 mM or 20 mM D-glucose (NG and HG groups, respectively) for 8 days, with or without 100 μM leflunomide, a positive modulator of Mfn2 [20] (cat. no. 14860; Cayman Chemical, Ann Arbor, MI, USA; HG/Lef). A group of HRECs were incubated for four days in 20 mM D-glucose, followed by four additional days in 5 mM D-glucose in a medium supplemented without or with leflunomide (HG-NG and HG-NG/Lef groups, respectively). HRECs incubated in 20 mM L-glucose (L-Gl) instead of 20 mM D-glucose were used in each experiment, and served as metabolic/osmotic control [16,17].

GTPase activities of Mfn2 were measured using a GTPase assay kit (DATG-200; Bioassay Systems, Hayward, CA, USA) in the immunoprecipitated samples, as previously described [17]. Release of phosphate was quantified spectrophotometrically at 620 nm, and the values were represented as -fold changes in phosphate release, considering the values obtained from HRECs in normal glucose as one.

Mitochondrial fragmentation was performed on coverslips via live cell imaging, as reported previously [16,17]. Cells were incubated with 200 nM MitoTracker Green FM (cat. no. M7514, Thermo Fisher Scientific, Waltham, MA, USA) for 10 min, and after washing the coverslips with PBS (3×), they were imaged under a Zeiss ApoTome fluorescence microscope (Carl Zeiss, Inc., Chicago, IL, USA) using a 40× objective. Mitochondrial fragmentation was performed in 3–5 coverslips/group from three different cell preparations.

Mitochondrial ROS levels were quantified by incubating HRECs with 5 μM MitoSOX, a mitochondrial superoxide indicator (cat. no. M36008, Thermo Fisher Scientific), in the presence of 200 nM MitoTracker Green for 30 min at 37 °C. Six to eight cells/group/experiment were imaged under a Zeiss Apotome at 20× magnification, and the intensity of the MitoSOX was quantified by ImageJ software (ImageJ, U.S. National Institutes of Health, Bethesda, MD, USA) [37]. Each group had at least three coverslips, and measurements were repeated in 3–4 different cell preparations. The ‘arithmetic mean intensity’ (AMI) was determined using the Zeiss colocalization software module (Carl Zeiss, Inc.) [38].

Mitochondrial respiration was determined by measuring OCR using a Seahorse XF analyzer (Agilent Technologies, Santa Clara, CA, USA). HRECs seeded in a 96-well cell culture plate were incubated in different experimental conditions and were washed 2× with 100 µL assay medium (Seahorse XF DMEM medium supplemented with 1 mM pyruvate, 2 mM glutamine and 10 mM glucose). This was followed by the addition of 180 µL assay medium in each well and incubation at 37 °C for 30 min. Using a Seahorse XF Cell Mito Stress Test Kit (cat. no. 103015-100, Agilent Technologies) and following the manufacturer’s protocol by injecting an ATP synthesis blocker oligomycin (1.5 µM), a mitochondrial uncoupler, carbonyl cyanide p-trifluoro methoxyphenylhydrazone (2.0 µM FCCP), and inhibitors of complex I and complex III, rotenone/antimycin A (0.5 µM) in ports A, B and C respectively, OCR was determined [39,40]. The data were collected and analyzed using Wave software (Agilent Technologies).

Mitochondrial DNA damage was determined by quantifying oxidatively modified DNA, 8-OHdG, using an ELISA kit (cat. no. 589320, Cayman Chemical) and following the manufacturer’s instructions, as reported previously [41]. Briefly, DNA (20 µg) was incubated for one hour with a monoclonal antibody against 8-OHdG in a microtiter plate precoated with 8-OHdG. The final color was developed by the addition of Ellman’s reagent and the absorbance was measured at 405 nm.

Mitochondrial DNA damage was also assessed in the genomic DNA using extended-length PCR. Long (8.8 kb) and short (223 bp) amplicons for mtDNA’s D-loop region were amplified, and the products were resolved on 1.2% agarose gel; a decrease in the ratio of long to short amplicons indicated damage to the mtDNA. Mitochondrial DNA damage was also confirmed by quantifying mRNA levels of mtDNA-encoded cytochrome b (*CytB)* of complex III of the electron transport chain using qRT-PCR [21,37].

Mitophagy flux was measured in HRECs using an Mtphagy kit (cat. no. MD01-10, Dojindo Molecular Technologies, Rockville, MD, USA). Briefly, HRECs were washed with DMEM and incubated with 100 nM Mtphagy Dye (diluted in DMEM) for 30 min at 37 °C. This was followed by washing the cells with DMEM and then trypsinizing them. After washing the cells (2×) with FACS buffer (PBS containing 0.5% BSA), the fluorescence intensity of the Mtphagy Dye was quantified using flow cytometry under a PerCP Cy5.5 channel (excitation/emission = 486/679) [42]. Raw flow cytometry standard files were analyzed by FlowJo v10.8.1 software (BD Biosciences, San Jose, CA, USA). Relative Mtphagy dye scattering proportional to the amount of mitophagy was plotted.

Mitophagy was also confirmed in HRECs using fluorescence microscopy by staining cells with Mtphagy Dye and Lyso Dye. Briefly, cells were incubated with 100 nM Mtphagy Dye for 30 min, and after washing them with DMEM, cells were treated with 1 µM Lyso Dye for 15 min. After removing the excess dye by washing them with PBS (2×), cells were visualized under a Zeiss Apotome fluorescence microscope at 20× magnification. The fluorescence intensity of the Mtphagy Dye was calculated to measure the mitophagy level [43].

Since mitophagy is intimately associated with change in mitochondrial membrane potential, mitochondrial transmembrane potential was determined by a mitochondrial binding dye, JC-1 (cat. no. MP03168, Molecular Probes, Carlsbad, CA, USA). HRECs were washed with PBS and incubated in DMEM containing 5 μM JC-1 for 30 min at 37 °C. After washing them with PBS, they were visualized under a Zeiss Apotome fluorescence microscope at 20× magnification. The fluorescence intensity was measured by the ratio of the aggregates (red, at 525 nm excitation and 590 nm emission wavelengths) to the monomers (green, at 485 nm excitation and 530 nm emission wavelengths) using a Zeiss software module [37].

Mitochondrial biogenesis was determined by quantifying copy numbers and mitochondrial mass. Using genomic DNA, gene transcripts of mtDNA-encoded *CytbB* and nuclear DNA-encoded *β-actin* were quantified by qRT-PCR and the ratio of the gene transcripts of *CytB* and *β-actin* was calculated.

Mitochondria mass was determined using MitoTracker Green labeling by incubating the cells with 200 nm MitoTracker Green for 30 min; the stained cells were visualized under a Zeiss ApoTome using a 20× objective. The fluorescence intensity of the MitoTracker was calculated using a Zeiss software module [44].

Mitochondrial mass was also confirmed using flow cytometry [45]; HRECs labeled with 100 nM MitoTracker Green for 30 min at 37 °C were washed with PBS, trypsinized, and the fluorescence intensity of MitoTracker Green was measured in a flow cytometer. The data were analyzed using FlowJo v10.8.1 software.

Statistical analysis: Data are presented as mean ± standard deviation. Comparison between groups was made using one-way ANOVA, followed by the Dunn post hoc test, and a *p* value < 0.05 was considered significant.

## Data Availability

R.A.K. is the guarantor of this work, and as such had full access to all the data in the study and takes responsibility for the integrity of the data and the accuracy of the data analysis. The data presented in this study are available on request from the corresponding author.

## References

[1] Diabetes Control and Complications Trial Research Group (1993). The effect of intensive treatment of diabetes on the development of long-term complications in insulin-dependent diabetes mellitus. N. Engl. J. Med..

[2] Lachin J.M., White N.H., Hainsworth D.P., Sun W., Cleary P.A., Nathan D.M., Diabetes Control and Complications Trial (DCCT)/Epidemiology of Diabetes Interventions and Complications (EDIC) Research Group (2015). Effect of intensive diabetes therapy on the progression of diabetic retinopathy in patients with type 1 diabetes: 18 years of follow-up in the DCCT/EDIC. Diabetes.

[3] Hainsworth D.P., Bebu I., Aiello L.P., Sivitz W., Gubitosi-Klug R., Malone J., White N.H., Danis R., Wallia A., Gao X. (2019). Risk Factors for Retinopathy in Type 1 Diabetes: The DCCT/EDIC Study. Diabetes Care.

[4] White N.H., Sun W., Cleary P.A., Tamborlane W.V., Danis R.P., Hainsworth D.P., Davis M.D., DCCT-EDIC Research Team (2010). Effect of prior intensive therapy on 10-year progression of retinopathy in DCCT/EDIC: Comparison of adults and adolescents. Diabetes.

[5] Aiello L.P. (2014). Diabetic retinopathy and other ocular findings in the diabetes control and complications trial/epidemiology of diabetes interventions and complications study. Diabetes Care.

[6] Gubitosi-Klug R.A., Sun W., Cleary P.A., Braffett B.H., Aiello L.P., Das A., Tamborlane W., Klein R., Writing Team for the DCCT/EDIC Research Group (2016). Effects of Prior Intensive Insulin Therapy and Risk Factors on Patient-Reported Visual Function Outcomes in the Diabetes Control and Complications Trial/Epidemiology of Diabetes Interventions and Complications (DCCT/EDIC) Cohort. JAMA Ophthalmol..

[7] Engerman R.L., Kern T.S. (1987). Progression of incipient diabetic retinopathy during good glycemic control. Diabetes.

[8] Kowluru R.A. (2003). Effect of reinstitution of good glycemic control on retinal oxidative stress and nitrative stress in diabetic rats. Diabetes.

[9] Kowluru R.A., Zhong Q., Kanwar M. (2010). Metabolic memory and diabetic retinopathy: Role of inflammatory mediators in retinal pericytes. Exp. Eye Res..

[10] Mizutani M., Kern T.S., Lorenzi M. (1996). Accelerated death of retinal microvascular cells in human and experimental diabetic retinopathy. J. Clin. Investig..

[11] Spinelli J.B., Haigis M.C. (2018). The multifaceted contributions of mitochondria to cellular metabolism. Nat. Cell Biol..

[12] Kowluru R.A., Kowluru A., Mishra M., Kumar B. (2015). Oxidative stress and epigenetic modifications in the pathogenesis of diabetic retinopathy. Prog. Retin. Eye Res..

[13] Kowluru R.A., Mishra M. (2015). Oxidative stress, mitochondrial damage and diabetic retinopathy. Biochim. Et Biophys. Acta.

[14] Yu R., Lendahl U., Nistér M., Zhao J. (2020). Regulation of Mammalian Mitochondrial Dynamics: Opportunities and Challenges. Front. Endocrinol..

[15] Yapa N.M.B., Lisnyak V., Reljic B., Ryan M.T. (2021). Mitochondrial dynamics in health and disease. FEBS Lett..

[16] Duraisamy A.J., Mohammad G., Kowluru R.A. (2019). Mitochondrial fusion and maintenance of mitochondrial homeostasis in diabetic retinopathy. Biochim. Biophys. Acta Mol. Basis Dis..

[17] Mohammad G., Kowluru R.A. (2022). Mitochondrial Dynamics in the Metabolic Memory of Diabetic Retinopathy. J. Diabetes Res..

[18] Kowluru R.A., Chan P.S. (2010). Metabolic memory in diabetes-from in vitro oddity to in vivo problem: Role of apoptosis. Brain Res. Bull..

[19] Kowluru R.A., Mohammad G. (2020). Epigenetics and Mitochondrial Stability in the Metabolic Memory Phenomenon Associated with Continued Progression of Diabetic Retinopathy. Sci. Rep..

[20] Miret-Casals L., Sebastián D., Brea J., Rico-Leo E.M., Palacín M., Fernández-Salguero P.M., Loza M.I., Albericio F., Zorzano A. (2018). Identification of New Activators of Mitochondrial Fusion Reveals a Link between Mitochondrial Morphology and Pyrimidine Metabolism. Cell Chem. Biol..

[21] Madsen-Bouterse S.A., Mohammad G., Kanwar M., Kowluru R.A. (2010). Role of mitochondrial DNA damage in the development of diabetic retinopathy, and the metabolic memory phenomenon associated with its progression. Antioxid. Redox Signal..

[22] Lee Y., Lee H.Y., Hanna R.A., Gustafsson Å.B. (2011). Mitochondrial autophagy by Bnip3 involves Drp1-mediated mitochondrial fission and recruitment of Parkin in cardiac myocytes. Am. J. Physiol. Heart Circ. Physiol..

[23] Bouchez C., Devin A. (2019). Mitochondrial Biogenesis and Mitochondrial Reactive Oxygen Species (ROS): A Complex Relationship Regulated by the cAMP/PKA Signaling Pathway. Cells.

[24] Uittenbogaard M., Chiaramello A. (2014). Mitochondrial biogenesis: A therapeutic target for neurodevelopmental disorders and neurodegenerative diseases. Curr. Pharm. Des..

[25] Twig G., Shirihai O.S. (2011). The interplay between mitochondrial dynamics and mitophagy. Antioxid. Redox Signal..

[26] Tilokani L., Nagashima S., Paupe V., Prudent J. (2018). Mitochondrial dynamics: Overview of molecular mechanisms. Essays Biochem..

[27] Galloway C.A., Lee H., Yoon Y. (2012). Mitochondrial morphology-emerging role in bioenergetics. Free Radic. Biol. Med..

[28] Lee Y.J., Jeong S.Y., Karbowski M., Smith C.L., Youle R.J. (2004). Roles of the mammalian mitochondrial fission and fusion mediators Fis1, Drp1, and Opa1 in apoptosis. Mol. Biol. Cell.

[29] Cortassa S., Aon M.A., Winslow R.L., O’Rourke B. (2004). A mitochondrial oscillator dependent on reactive oxygen species. Biophys. J..

[30] Fragoso Y.D., Brooks J.B. (2015). Leflunomide and teriflunomide: Altering the metabolism of pyrimidines for the treatment of autoimmune diseases. Expert. Rev. Clin. Pharm..

[31] Maremanda K.P., Sundar I.K., Rahman I. (2021). Role of inner mitochondrial protein OPA1 in mitochondrial dysfunction by tobacco smoking and in the pathogenesis of COPD. Redox Biol..

[32] Moon S.J., Kim E.K., Jhun J.Y., Lee H.J., Lee W.S., Park S.H., Cho M.L., Min J.K. (2017). The active metabolite of leflunomide, A77 1726, attenuates inflammatory arthritis in mice with spontaneous arthritis via induction of heme oxygenase-1. J. Transl. Med..

[33] Pellattiero A., Scorrano L. (2018). Flaming Mitochondria: The Anti-inflammatory Drug Leflunomide Boosts Mitofusins. Cell Chem. Biol..

[34] Abdel-Hamid N.M., Abass S.A., Eldomany R.A., Abdel-Kareem M.A., Zakaria S. (2022). Dual regulating of mitochondrial fusion and Timp-3 by leflunomide and diallyl disulfide combination suppresses diethylnitrosamine-induced hepatocellular tumorigenesis in rats. Life Sci..

[35] Yu M., Nguyen N.D., Huang Y., Lin D., Fujimoto T.N., Molkentine J.M., Deorukhkar A., Kang Y., San Lucas F.A., Fernandes C.J. (2019). Mitochondrial fusion exploits a therapeutic vulnerability of pancreatic cancer. JCI Insight.

[36] Anzell A.R., Maizy R., Przyklenk K., Sanderson T.H. (2018). Mitochondrial Quality Control and Disease: Insights into Ischemia-Reperfusion Injury. Mol. Neurobiol..

[37] Mohammad G., Kowluru R.A. (2021). Nuclear genome-encoded long noncoding RNAs and mitochondrial damage in diabetic retinopathy. Cells.

[38] Kumar J., Mohammad G., Alka K., Kowluru R.A. (2022). Mitochondrial Genome-Encoded Long Noncoding RNA and Mitochondrial Stability in Diabetic Retinopathy. Diabetes.

[39] Navneet S., Zhao J., Wang J., Mysona B., Barwick S., Ammal Kaidery N., Saul A., Kaddour-Djebbar I., Bollag W.B., Thomas B. (2019). Hyperhomocysteinemia-induced death of retinal ganglion cells: The role of Muller glial cells and NRF2. Redox Biol..

[40] Thounaojam M.C., Jadeja R.N., Warren M., Powell F.L., Raju R., Gutsaeva D., Khurana S., Martin P.M., Bartoli M. (2019). MicroRNA-34a (miR-34a) Mediates Retinal Endothelial Cell Premature Senescence through Mitochondrial Dysfunction and Loss of Antioxidant Activities. Antioxidants.

[41] Kowluru R.A., Menon B., Gierhart D.L. (2008). Beneficial effect of zeaxanthin on retinal metabolic abnormalities in diabetic rats. Investig. Ophthalmol. Vis. Sci..

[42] Lin Y.C., Lin Y.C., Tsai M.L., Liao W.T., Hung C.H. (2022). TSLP regulates mitochondrial ROS-induced mitophagy via histone modification in human monocytes. Cell Biosci..

[43] Zhang Y., Wang S., Chen X., Wang Z., Wang X., Zhou Q., Fang W., Zheng C. (2022). Liraglutide prevents high glucose induced HUVECs dysfunction via inhibition of PINK1/Parkin-dependent mitophagy. Mol. Cell. Endocrinol..

[44] Santos J.M., Tewari S., Goldberg A.F.X., Kowluru R.A. (2011). Mitochondria biogenesis and the development of diabetic retinopathy. Free Radic. Biol. Med..

[45] Hegedűs C., Boros G., Fidrus E., Kis G.N., Antal M., Juhász T., Janka E.A., Jankó L., Paragh G., Emri G. (2019). PARP1 Inhibition Augments UVB-Mediated Mitochondrial Changes-Implications for UV-Induced DNA Repair and Photocarcinogenesis. Cancers.

